# Multidetector CT of expected findings and early postoperative complications after current techniques for ventral hernia repair

**DOI:** 10.1007/s13244-016-0501-x

**Published:** 2016-05-18

**Authors:** Massimo Tonolini, Sonia Ippolito

**Affiliations:** Department of Radiology, “Luigi Sacco” University Hospital, Via G.B. Grassi 74, 20157 Milan, Italy

**Keywords:** Ventral hernia, Hernioplasty, Prosthetic mesh, Complications, Computed tomography (CT)

## Abstract

**Abstract:**

Current techniques for ventral hernia repair (VHR) rely on prosthetic mesh implantation and are increasingly performed laparoscopically. Potentially serious iatrogenic complications may occur following VHR, though these are rare compared to the vast number of procedures performed each year. This paper provides an overview of contemporary open and laparoscopic surgical techniques and biomaterials, then reviews and illustrates the expected postoperative imaging appearances, and common and unusual early complications after VHR. Emphasis is placed on multidetector computed tomography (CT), which comprehensively visualizes the operated anterior abdominal wall and deeper intra-abdominal structures. CT consistently allows diagnosis of postoperative seromas, abdominal wall abscesses and fistulas, haemorrhages with or without active bleeding, bowel obstruction, peritonitis and recurrent hernias, and thus providing a reliable basis for an appropriate choice between conservative, interventional, or surgical treatment. Familiarity with early post-surgical CT is warranted to avoid misinterpretation of the expected imaging appearance and correctly elucidate postoperative complications after VHR.

***Teaching points*:**

• *Open and laparoscopic repair of ventral hernias rely on prosthetic mesh implantation.*

• *Potentially serious iatrogenic complications occasionally occur after ventral hernioplasty.*

• *Multidetector CT consistently evaluates the operated abdominal wall and deeper structures.*

• *Familiarity with the expected early postoperative imaging appearance is required.*

• *Complications include seroma, infections, haemorrhage, bowel obstruction, peritonitis, and recurrence.*

## Introduction

Since the early 1990s, two major technical advances have revolutionized the field of ventral hernia repair (VHR). Firstly, most surgeons switched from traditional to modern mesh-based “tension-free” techniques, thus achieving a significant decrease in the rate of hernia recurrence [1, 2]. Secondly, despite requiring greater expertise and a longer operation time, the increasing use of laparoscopy in VHR has allowed better cosmetic results and faster return to normal activities [3, 4].

Rarely, in a minority of operated patients, VHR results in potentially serious short-term complications. Owing to the vast number of open and laparoscopic interventions performed each year at most general hospitals, radiologists may be confronted with urgent requests to investigate suspected iatrogenic injuries. However, the radiological reports on this subject that are currently available are mostly focused on the normal imaging appearance of prosthetic meshes (PMs), the occurrence and fate of common post-surgical seromas [5, 6], the value of computed tomography (CT) for diagnosis of recurrent hernias [7], and complications secondary to laparoscopic access for different surgical procedures [8].

Conversely, very limited literature is available describing the imaging appearance of common and rare postoperative complications after VHR. This pictorial essay provides an overview of curent open and laparoscopic surgical techniques, then reviews and illustrates the expected postoperative appearance and the imaging features of iatrogenic complications. Most emphasis is placed on multidetector CT, which represents the mainstay modality to promptly and comprehensively visualize the operated abdominal wall and deeper intra-abdominal structures, thus providing a consistent basis for appropriate choice between conservative, interventional, or surgical treatment. Since timely recognition and management of complications are essential in limiting iatrogenic morbidity, the aim of this pictorial essay is to improve radiologists’ familiarity with interpretation of early post-surgical CT studies.

## Overview of surgical techniques

Broadly defined as “a protrusion of tissues through a defect of the anterior abdominal wall”, ventral hernias represent a highly prevalent but heterogeneous problem in general surgery. The European Hernia Society (EHS) categorizes abdominal wall hernias as either primary or incisional. The former group includes midline (epigastric and umbilical) and lateral (spigelian and lumbar) hernias, which are classified according to their size (<2 cm, >4 cm, or intermediate). Congenital or acquired umbilical hernias represent the most common primary hernias [9].

An incisional hernia is defined as “any abdominal wall gap with or without a bulge in the area of a postoperative scar perceptible or palpable by clinical examination or imaging from incisional hernias”. Incisional hernias develop after any abdominal surgery, with an incidence approaching 20 %, and are increasingly encountered because of increased life expectancy and the high prevalence of risk factors such as obesity and diabetes. Recurrences after repair of primary hernias fall in the incisional group [9].

Indications for VHR include pain, cosmesis, and prevention or treatment of complications such as bowel obstruction and strangulation [1, 2]. At our hospital, the mean case volume approaches 110 VHR procedures each year; the vast majority (85–90 %) are for incisional hernias.

Before the availability of modern PMs, the rate of postoperative hernia recurrence approached 50 %. Current techniques for VHR rely on PM implantation, may be performed during either open surgery or – increasingly – laparoscopic surgery, and consistently achieve favourable results with minimal perioperative morbidity (3.7 % overall complication rate) and limited (7.5–10 %) recurrence rate [1, 3, 4, 10–12].

Open VHR is recommended for large (>10 cm) abdominal wall defects. The sublay-mesh (Rives–Stoppa) procedure remains the preferred open technique to treat ventral (particularly incisional) hernias. Following skin incision, the surgeon opens the hernial sac, dissects the anterior abdominal structures up to the preperitoneal space, and removes the previous surgical scar, adhesions in the abdominal cavity and the hernial sac itself. The PM is then implanted extraperitoneally in the newly formed space, interposed between the posterior rectus sheath and rectus muscle. Very large hernias with lateral displacement of the rectus abdominis muscles require open repair with the intraperitoneal onlay-mesh (IPOM) technique: after exposure and opening of the hernial sac, adhesions between the abdominal wall and intestinal loops are detached, the hernial sac is removed, and a large PM is positioned from the inside over the breach [10–14].

Conversely, laparoscopic VHR involves creation of pneumoperitoneum, introduction of instruments through the trocar ports, and reduction of herniated bowel loops into the peritoneal cavity. The PM is placed intraperitoneally over the defect, without excision of the hernia sac [15].

With either approach, sutures, fixation screws or an additional second PM may be used to reinforce the repair. Thorough yet easy-to-understand step-by-step graphical explanations of the open and laparoscopic techniques described above are freely downloadable from the dedicated www.herniamed.de website [13–15].

## Imaging techniques after ventral hernia repair

### Role of ultrasonography

After VHR, the early postoperative assessment is essentially based on physical findings. Some authors have advocated ultrasonography as a useful adjunct to clinical evaluation. Although sonographic visualization of the PM is inconsistent, ultrasonography may rapidly detect anechoic collections of serous fluid and variably echogenic haematomas [16, 17]. However, particularly in the early postoperative setting, physical and sonographic assessment are both frequently hampered by obesity, thickened subcutaneous fat, the presence of medications, and local tenderness at the surgical wound.

### Multidetector CT: role and technique

Compared to ultrasonography, cross-sectional imaging with multidetector CT consistently provides panoramic visualization of normal structures and postoperative changes at the anterior abdominal wall, and often provides additional information that may prove crucial for identification of possible complications [5–8, 18].

Similarly to the preoperative assessment of ventral hernias, the CT acquisition protocol should encompass the entire abdomen, from the diaphragm to the symphysis pubis. Strategies for dose reduction, such as automated tube current modulation, or, if available, iterative reconstruction, are recommended [19].

Unless contraindicated by allergy or renal failure, contrast enhancement is warranted when there is concern about infection, haemorrhage, or bowel complication. In most patients, we routinely obtain a portal-venous phase acquisition, which, in our experience, provides the best delineation of seromas, fistulas, and abscesses. A preliminary unenhanced scan allows identification and measurement of the size and Hounsfield unit (HU) attenuation of fluid or haemorrhagic collections, but may be omitted in order to limit the dose of ionizing radiation, particularly in patients who are younger than 45 years. When clinically suspected, an additional arterial-phase scanning is beneficial to detect active bleeding. In addition to reviewing axial images, we recommend routine image reconstruction and study interpretation along the sagittal orientation, which provides the best visualization of the anterior abdominal wall [5, 8, 18].

## Biomaterials used for ventral hernia repair

A proliferation of mesh products for VHR are currently available. The most common PMs are made of polypropylene (PP) or expanded polytetrafluoroethylene (ePTFE). Both types are invisible on plain radiographs, and sonographically appear hyperechoic with posterior acoustic shadowing. Visualization of PMs by CT is highly variable, depending on the intrinsic density, thickness, woven or nonwoven, knitted or not-knitted structure, and surrounding inflammatory reaction [20].

**Fig. 1:**
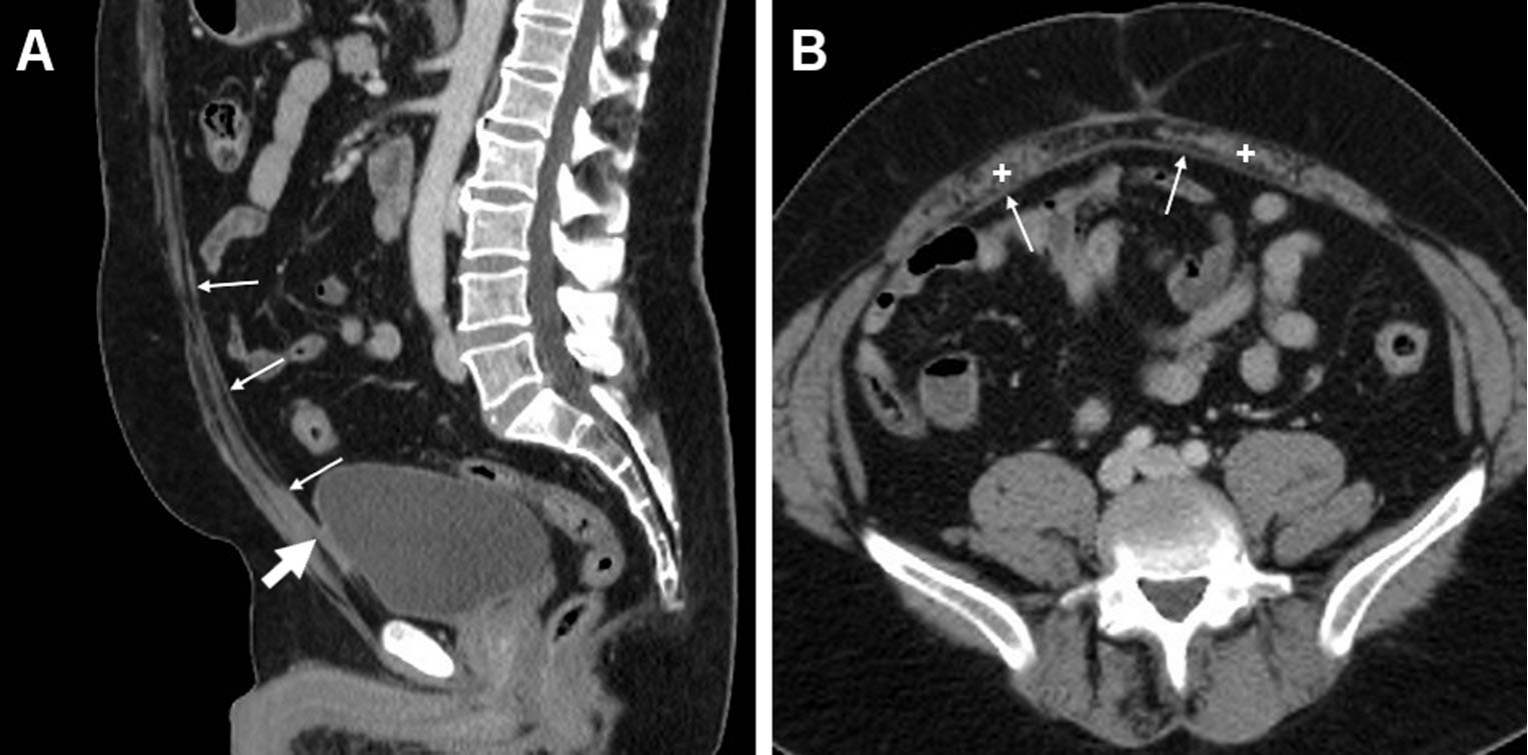
Usual imaging appearance of polypropylene (PP) prosthetic mesh (PM) in a 72-year-old male, two weeks after uncomplicated open repair (Rives-Stoppa technique) of a large ventral incisional hernia. On sagittal (**a**) and axial (**b**) post-contrast CT images, a thin structure (thin arrows) with soft-tissue attenuation is identified at the deep aspect of the atrophic rectus abdominis muscles (+), which corresponds to the PP PM interposed between the parietal peritoneum and muscle sheath. Asymptomatic adhesion of the caudalmost portion of the PM to the urinary bladder (thick arrow in A) was incidentally noted

**Fig. 2:**
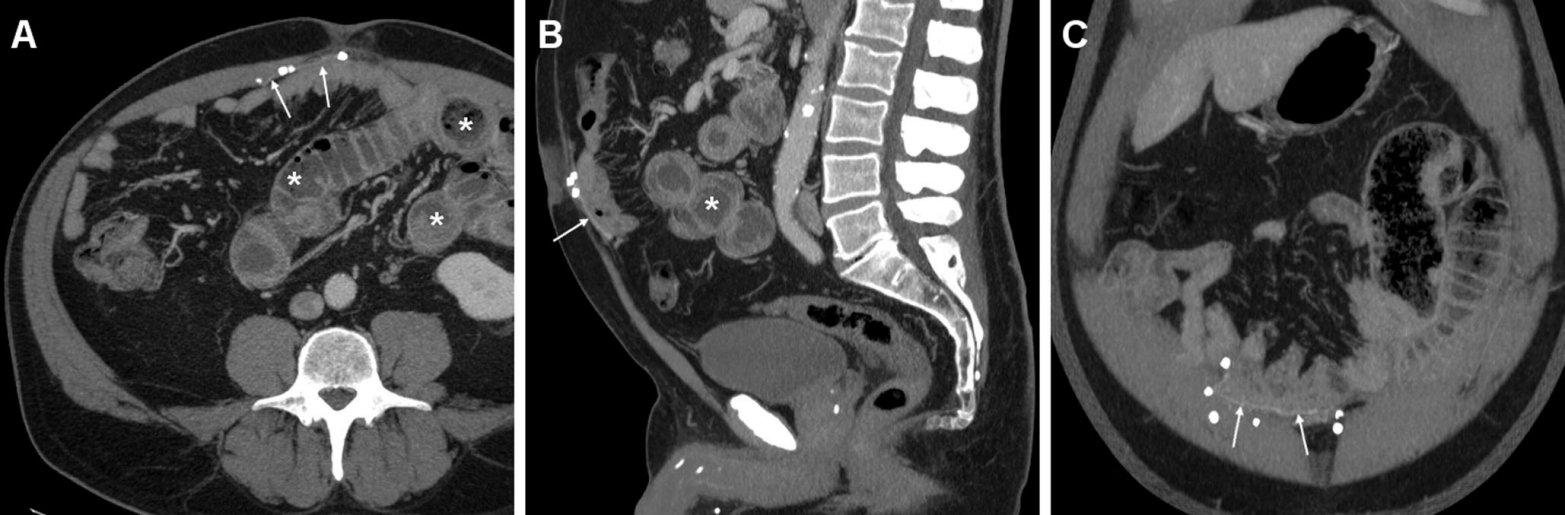
Usual imaging appearance of expanded polytetrafluorethylene (ePTFE) PM following recent laparoscopic repair of a ventral incisional hernia in a 70-year-old overweight male, suffering from postoperative abdominal pain and vomiting. Axial (**a**), sagittal (**b**) contrast-enhanced CT images and coronal maximum-intensity projection (MIP) reconstruction (**c**) showed the moderately hyperattenuating PM (thin arrows), fixed by metallic sutures, without abnormal collections. Note distended small bowel loops (*) with intraluminal fluid consistent with clinical diagnosis of postoperative intestinal obstruction, which required laparotomic surgery including redo hernia repair

**Fig. 3:**
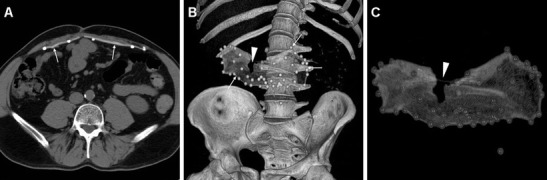
CT assessment of a 15x9 cm Gore-Tex Dual-Mesh® PM positioned laparoscopically 18 months earlier after previous failed attempts to repair an epigastric hernia. The ePTFE PM (thin arrows) secured by metallic-attenuation sutures is easily recognized on unenhanced axial CT image (**a**), and well depicted in its entirety and reticular structure by three-dimensional volume-rendering reconstructions (**b**, **c**). Additionally, a clinically unsuspected focal breach (arrowheads) was noted at its upper aspect, probably resulting from excessive tension

Monofilament, double-filament (such as Prolene; Ethicon, Inc., Somerville NJ-USA), and multifilament (such as Surgipro; Covidien, Minneapolis MN-USA) PP PMs are thin and isoattenuating to muscles and, therefore, generally invisible or hardly differentiated from the deeper fascia of the rectus abdominis muscles and parietal peritoneum (Fig. 1). Conversely, thicker ePTFE PMs (such as the Gore-Tex Dual-Mesh; W.L.Gore & Associates, Inc., Flagstaff, AZ–USA) are consistently recognized as hyperdense lines behind the muscles of the abdominal wall (Figs. 2 and 3). Finally, composite PMs including an ePTFE component (such as the Bard Composix; Bard Davol Inc., Warwick RI-USA) are less consistently recognizable. Visualization of faintly hyperattenuating PMs may be improved by the use of maximum-intensity projection (MIP) reconstructions (Fig. 2c). When present, metallic surgical staples or tacks used for mesh fixation are easily visible (Figs. 2 and 3). Additionally, three-dimensional volume-rendering images effectively visualize the position, spatial configuration, and reticular structure of the PM (Fig. 3) [5, 6, 8].

## Early postoperative CT findings and seromas

In the early postoperative setting, suction drains may be in place, and their position should be reported. Hours or a few days after recent laparoscopic VHR, some residual intraperitoneal gas is commonly observed. Similarly, mild to moderate subcutaneous emphysema from insufflation into the abdominal wall during trocar passage generally does not represent a complication, provided there are no clinical or laboratory findings suggesting peritonitis or necrotizing fasciitis [8].

Postoperative seromas develop at the operated anterior abdominal wall in almost 10 % of patients. After open VHR, serous fluid collects in the retromuscular–prefascial space; conversely, following laparoscopic surgery, seromas result from peritoneal fluid flowing through the porous PM and accumulating within the residual hernia sac [8, 11, 12, 21].

**Fig. 4:**
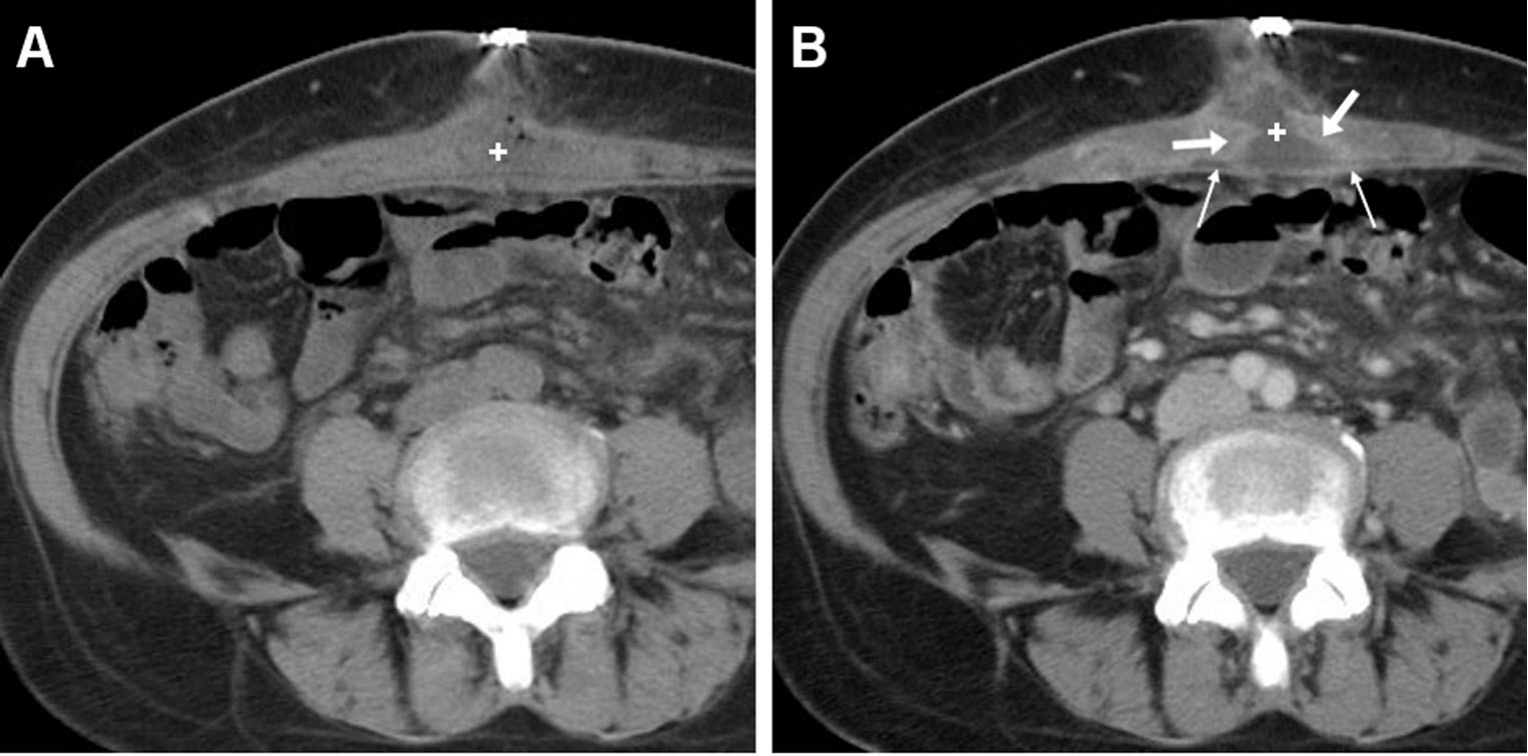
Common appearance of postoperative seroma detected six days after open ventral hernia repair (VHR) in a 51-year-old female. Unenhanced (**a**) and post-contrast (**b**) axial CT images showed a small-sized midline fluid-attenuating collection (+) containing tiny gas bubbles, interposed between the rectus muscles, abutting the PM-reinforced parietal peritoneum indicated by a linear structure isoattenuating to muscles (thin arrows). In the absence of clinical and laboratory signs of infection, thin peripheral contrast enhancement (arrows in **b**) was observed. The seroma eventually resolved without any additional treatment

At CT, serous collections may have different shapes (globular, multiloculated, or tubular). Seroma is confidently diagnosed when a collection shows fluid attenuation and does not enhance. Unfortunately, in the early postoperative days, identification of air-fluid levels and thin reactive peripheral enhancement (Fig. 4) was not unusual. In this case, differentiation from an abscess is challenging and relies on correlation with clinical and laboratory findings. Conversely, a thick or irregular enhancing “rim” should suggest infection rather than seroma [5, 6, 8].

Seromas should be managed conservatively unless they are large, painful, or persistent after 4–6 weeks, and aspiration is contraindicated to prevent superinfection [3, 4, 8, 21].

## The appearance of complications on multidetector CT imaging

Although post-VHR iatrogenic complications are reported in up to 18–25 % of patients overall, the majority of cases are minor and do not require hospitalization or specific treatment. The commonest specific complications include infection, small bowel obstruction, and haemorrhage, in descending order of frequency [3, 4, 11, 12].

### Wound and deep infections

Wound infections occur more frequently after open VHR (9–14 % incidence) compared with laparoscopic surgery (<2 % of patients), and generally resolve with local wound care and washing and antibiotics. The more serious deep PM-related infections are reported in up to 2–4 % of patients, and are often associated with specific risk factors, including obesity, diabetes, and urgency, or prolonged duration of surgery. Clinical manifestations such as purulent discharge, tender swelling, fever, abnormal acute-phase reactants, and leukocyte count should be investigated with bacteriological cultures and treated aggressively. Mesh infection is of concern to surgeons, since it cannot be treated with antibiotics and almost invariably requires removal of the PM [2–4, 11, 12].

**Fig. 5:**
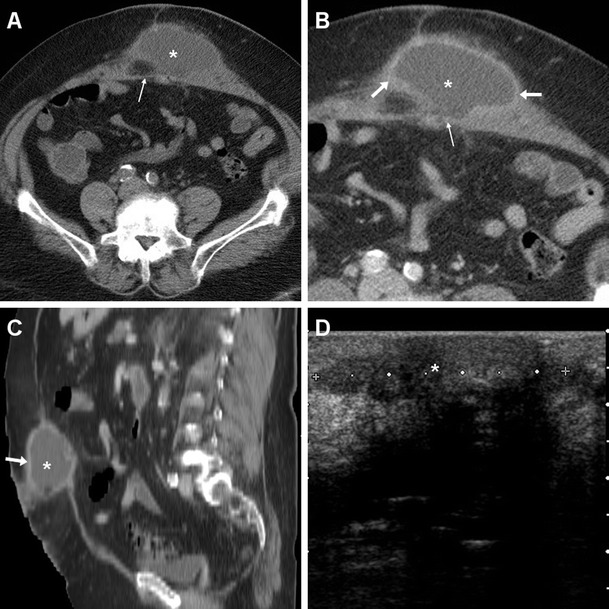
Postoperative abscess following recent open repair of post-laparotomic hernia in a 77-year-old female, clinically heralded by hard-consistency and inflamed swelling at the anterior abdominal wall. Unenhanced (**a**), post-contrast (**b**) axial and sagittal (**c**) CT images showed a sizeable (12x4 cm) collection (*) abutting the anterior aspect of the PM (thin arrows), with 16–18 Hounsfield units (HU) internal attenuation and thick irregular peripheral enhancement (arrows in **b**, **c**). The corresponding, unspecific sonographic appearance (**d**) was a poorly demarcated hypoechoic region (calipers) with posterior acoustic shadowing. The abscess resolved with percutaneous drainage and antibiotics

**Fig. 6:**
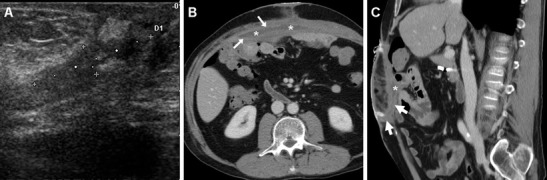
A 51-year-old male experienced wound dehiscence with culture-proven *Staphylococcus aureus* infection following Rives-Stoppa repair of a ventral incisional hernia. Ultrasound (**a**) showed an ill-defined, inhomogeneously hypoechoic region (caliper) at the surgical site, without fluid portions amenable to aspiration. Corresponding post-contrast axial (**b**) and sagittal (**c**) CT images showed a band-shaped abscess (*) of the anterior abdominal wall with enhancing periphery (arrows), interposed between the PM and rectus muscles, which drained externally through a fistula (thick arrows in **c**) (note hyperattenuating plug at skin orifice)

**Fig. 7:**
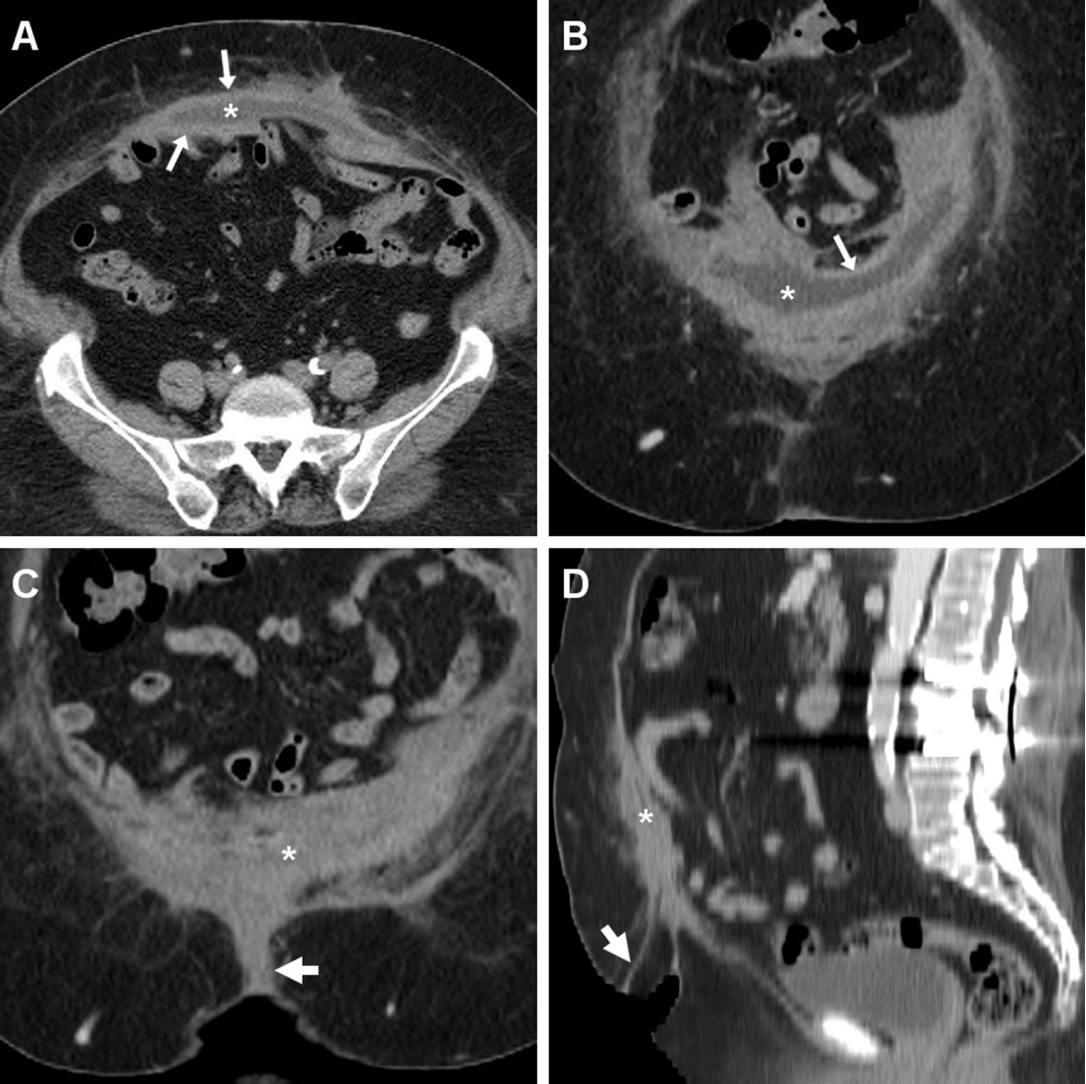
In a 75-year-old female, after recent open repair of post- hysterectomy incisional hernia, axial (**a**), coronal (**b**), and oblique-coronal (**c**) multidetector CT images depicted a crescent-shaped abscess of the anterior abdominal wall (*) with enhancing periphery, connected to the cutaneous opening by a fistula (thick arrows). Surgical reintervention with PM removal and replacement was required

**Fig. 8:**
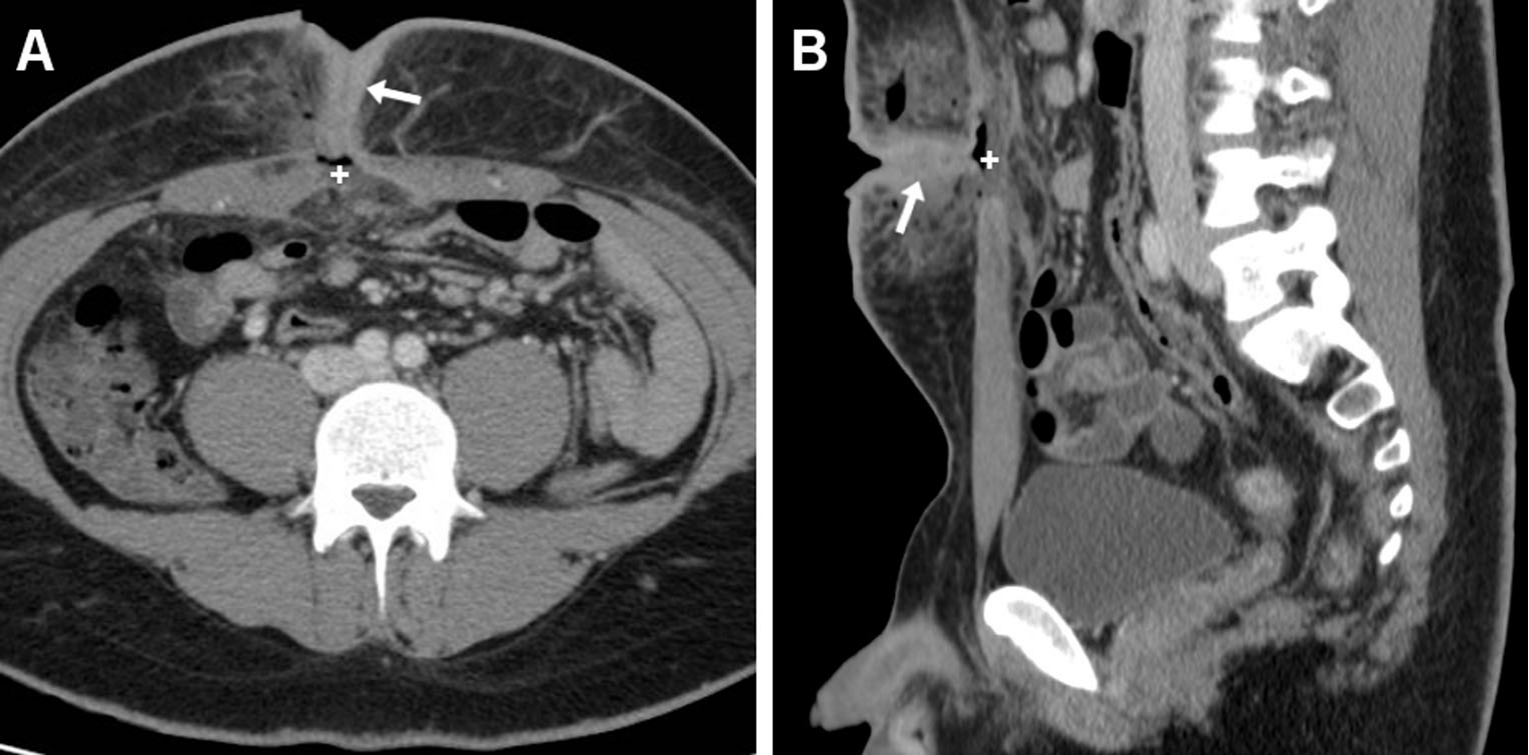
Another case of fistulising post surgical infection in a 41-year-old male after VHR of recurrent incisional hernia. Pus-yielding wound infection corresponded on axial (**a**) and sagittal (**b**) post-contrast CT images to a small-sized collection with mixed air (+) and fluid (*) content in the typical midline site, abutting the PM ventrally. A characteristic tram-track fistula (thick arrows) drained the collection towards the skin, and was a surrounded by extensive inflammatory stranding of the subcutaneous fat

The characteristic CT appearance of a post-surgical septic collection after VHR is that of an abscess with thick, irregular peripheral enhancement, which develops ventrally to the PM-reinforced parietal peritoneum, and may remain contained within the muscles of the abdominal wall (Fig. 5), or extend to the anterior abdominal skin through a cutaneous fistula (Figs. 6, 7 and 8).

### Haemorrhage

After VHR, abdominal wall haematomas may occur in up to 4.7 % of patients, resulting from unrecognized intraoperative injury to tiny vessels or to laparoscopic trocar placement. The variable clinical manifestations include swelling, pain, and ecchymosis. External bleeding from the trocar access port is not unusual, particularly in delayed bleeding [3, 4, 11, 12].

**Fig. 9:**
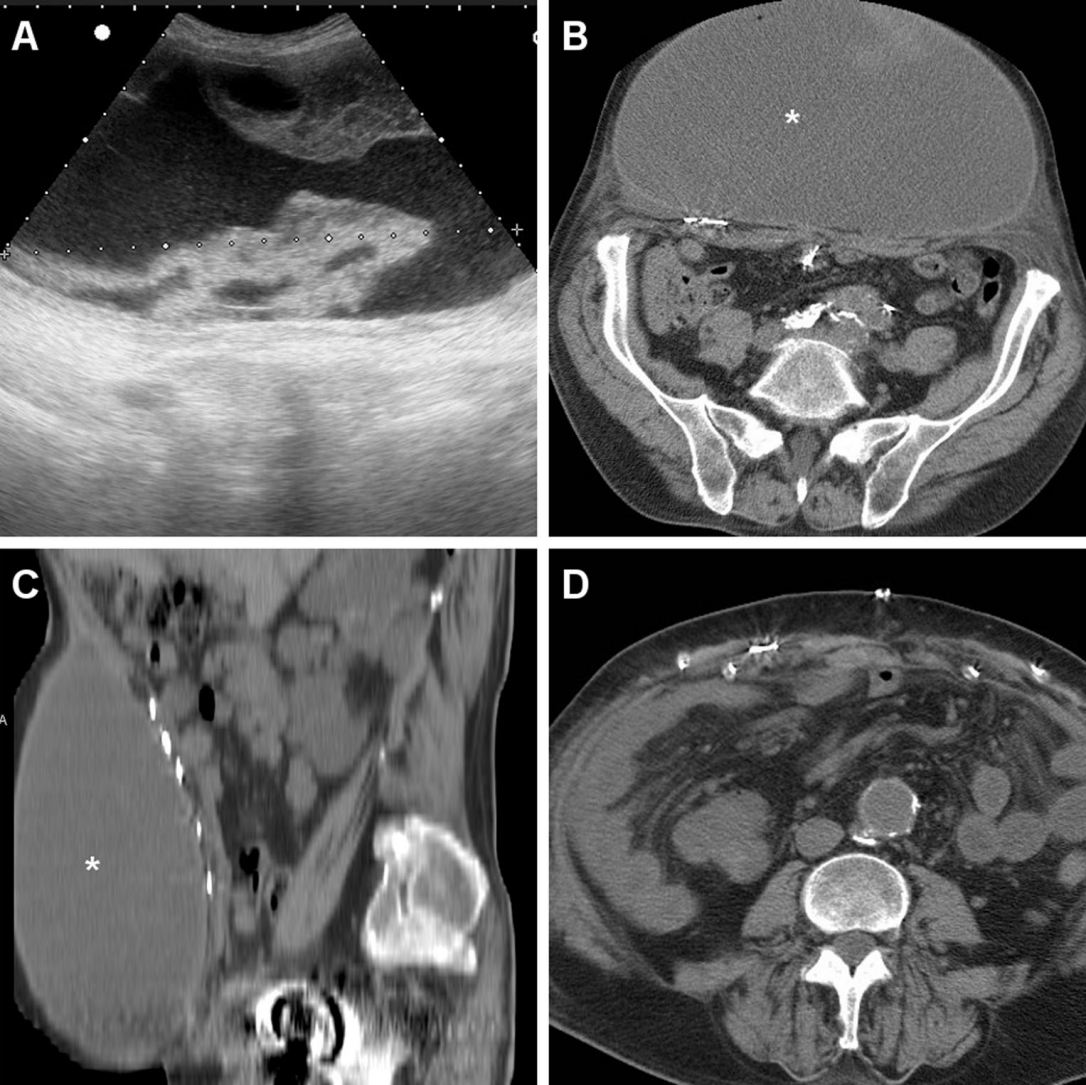
In a 75-year-old male, six weeks after VHR ultrasound (**a**) depicted a huge (25x11x18 cm) subacute haemorrhagic collection with mixed anechoic fluid, thick walls, and septa. Axial (**b**) and sagittal (**c**) unenhanced CT images depicted the liquefied haematoma (*) centered in the anterior abdominal wall, causing dislocation and compression of the PM indicated by the presence of metallic fixation clips. Repeated unenhanced CT (**d**) showed the postoperative status following drainage of two litres of serum and old blood

**Fig. 10:**
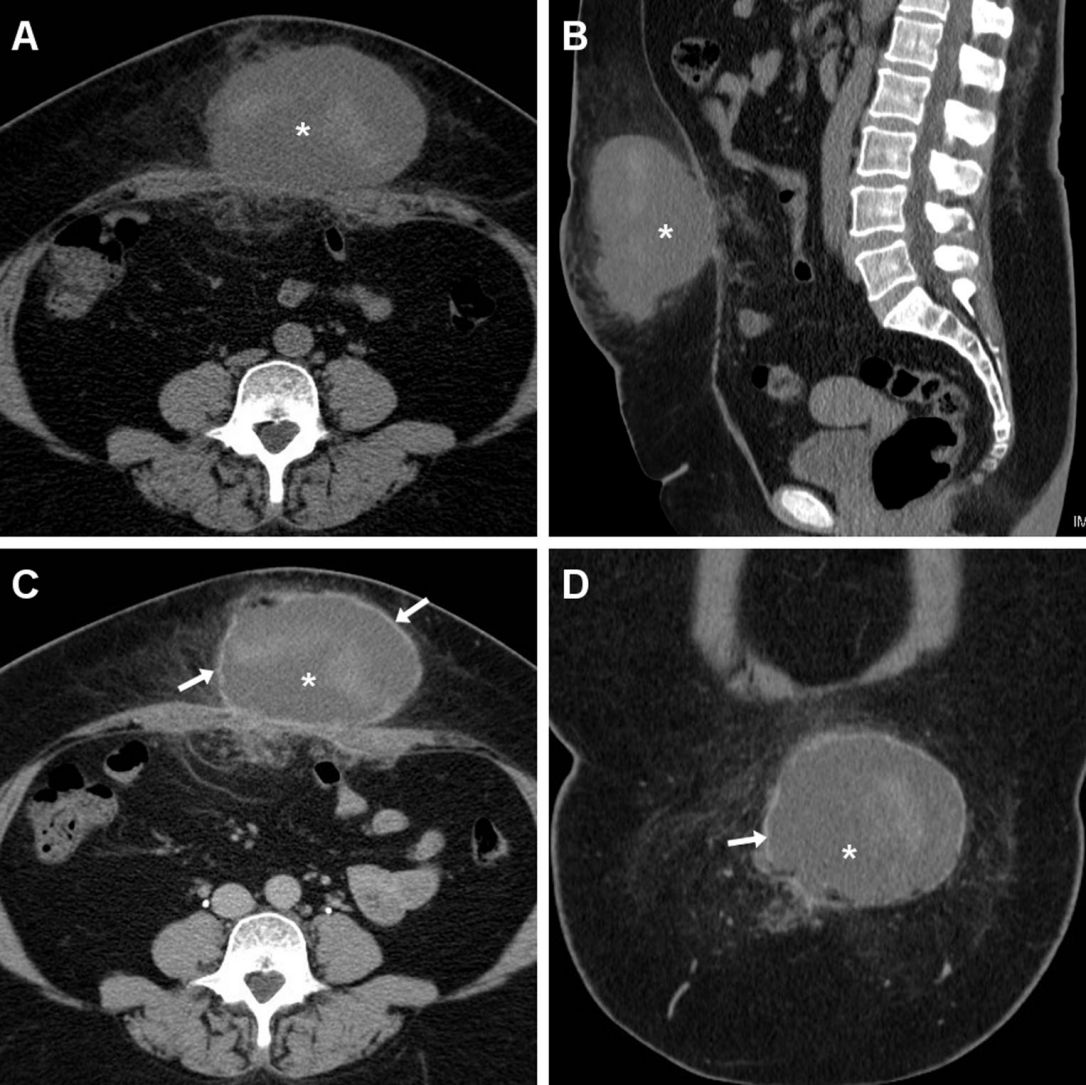
Post-surgical haematoma developed within three days after VHR in a 44-year-old female, with physical finding of postoperative swelling at the anterior abdominal wall. Unenhanced axial (**a**) and sagittal (**b**) images showed a demarcated midline collection with heterogeneous attenuation (*) abutting the rectus muscles externally and occupying the subcutaneous fat. Axial (**c**) and coronal (**d**) post-contrast CT images showed minimal peripheral enhancement (arrows), without signs of active bleeding

**Fig. 11:**
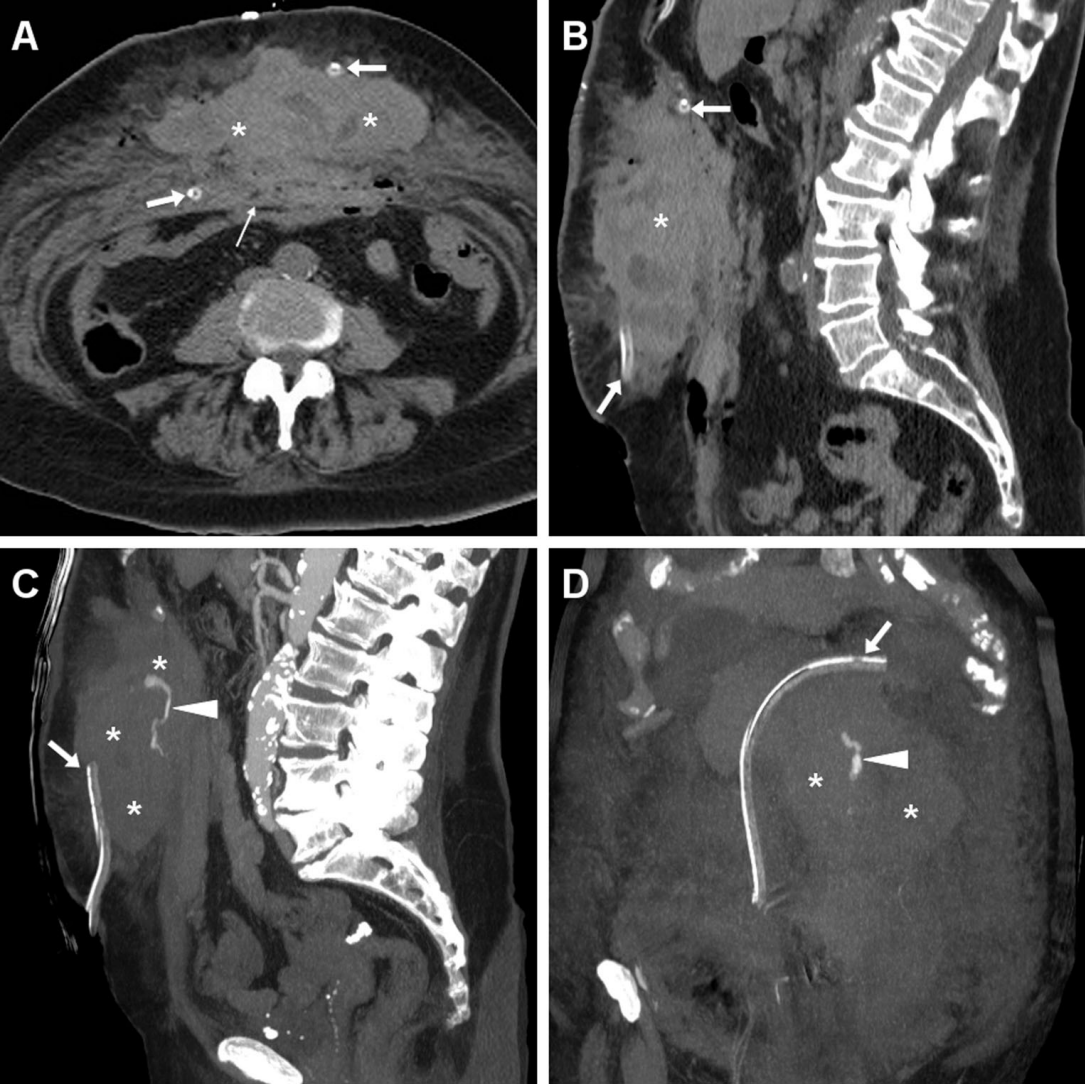
Active post-surgical bleeding in an elderly 84-year-old female with acute abdominal pain, hard-consistency swelling, and severe blood loss (8 g/dl nadir haemoglobin) 24 hours after Rives-Stoppa repair of a midline incisional hernia. Emergency CT depicted a 15x6x13 cm hyperattenuating (median 55 HU) fresh haematoma (*), extending ventrally from the surgical site through the rectus muscles and subcutaneous fat. Note PM (thin arrows) and drainage tubes (arrows). Sagittal (**c**) and coronal (**d**) MIP reconstructions showed serpiginous contrast extravasation (arrowheads) isoattenuating with the enhanced aortic lumen within the haematoma, consistent with active arterial bleeding. Relaparotomy confirmed bleeding suprafascial haematoma, which required haemostasis and surgical drainage [Partly reproduced with permission from Ref. no. [18]]

Multidetector CT reliably depicts abdominal wall haematomas in their entire extent, as high-density collections with attenuation ranging between 30 and 80 HU, depending on the duration of the bleeding (Figs. 9, 10, and 11). Surgical revision and PM removal are necessary when the haematocrit drops and CT findings indicate major bleeding. Although uncommon, the identification of active bleeding as contrasted with extravasation isoattenuating with enhanced vessels (Fig. 11) indicates the need for interventional or surgical treatment [8, 18].

### Bowel obstruction

Whereas postoperative ileus is rather common, in patients with clinical or radiographic signs of intestinal obstruction after VHR (Fig. 2), adhesions between the PM and the small (or occasionally the large) bowel should be considered as the most likely cause. In laparoscopic VHR, contiguity of bowel loops and intraperitoneally placed PM may ease the formation of adhesions. Alternatively, CT studies should be scrutinized for small-bowel herniation in a peritoneal breach, most usually a trocar access port. Conservative in-hospital treatment, including decompression by nasogastric tube, may be successful in relieving low-grade obstruction. Alternatively, laparotomic surgical revision with or without bowel resection may be required [8].

### Bowel injury

Although exceptional (0.06–0.2 % incidence), catastrophic complications such as iatrogenic bowel injury (IBI) occur more frequently after laparoscopic VHR. When unrecognized during surgery, bowel perforation manifests with fever, abdominal pain, and peritonitis within a week after VHR. Delayed recognition usually results in increased morbidity and mortality, with enterocutaneous fistula formation and sepsis [3, 4, 8].

**Fig. 12:**
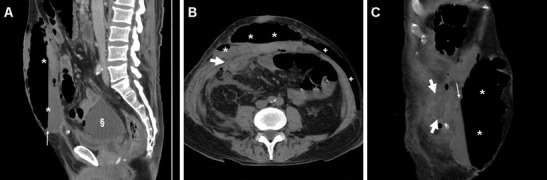
Surgically confirmed iatrogenic injury to the small bowel from laparoscopic VHR in a 76-year-old male. Sagittal (**a**), axial (**b**), and oblique coronal (**c**) images from contrast-enhanced early postoperative multidetector CT showed abundant fluid (§) and some air in the peritoneal cul-de-sac consistent with peritonitis, in communication (thick arrows) with a vast collection (*) with mixed content and air-fluid level (thin arrows) at the anterior abdominal wall

Moderate degrees of pneumoperitoneum are commonly observed during the first postoperative days after laparoscopic surgery. Conversely, CT evidence of peritonitis (Fig. 12) or abnormal intra-abdominal air-fluid collections should suggest the possibility of an IBI. Perforations usually require prompt surgery, including bowel resection or Hartmann’s procedure [8].

### Hernia recurrence

**Fig. 13:**
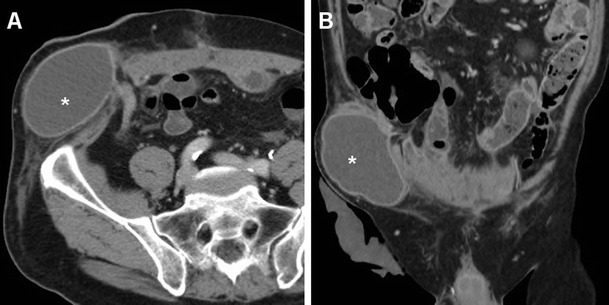
Failed repair of peristomal hernia in a 71-year-old male with a history of previous Bricker radical cystectomy, suffering from postoperative vomiting, persistent abdominal pain, and local swelling. Axial (**a**) and coronal (**b**) contrast-enhanced CT images showed recurrent herniation at the urostomy site of a dilated, fluid-filled structure consistent with the ileal conduit (*). Repeated surgery was performed

**Fig. 14:**
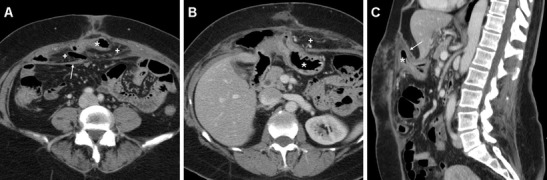
Failed repair of large ventral incisional hernia in a 50-year-old obese female. Four days after surgery, urgent CT was obtained because distended, tender abdomen revealed detachment of the PM (thin arrows) from the anterior abdominal wall, and recurrent ventral herniation of intra-abdominal fat (+) and of a portion of the gastric antrum (*)

Despite the considerably decreased incidence of recurrence afforded by tension-free repair, after either open or laparoscopic VHR, up to 8–9 % of patients still develop recurrent incisional hernias after a variable time interval [3, 4, 11, 12, 21]. In the vast majority of cases, hernia recurrence is reliably identified using CT (Figs. 13 and 14) [7].

## Conclusion

Multidetector CT allows comprehensive assessment of the operated anterior abdominal wall and is, therefore, recommended to elucidate suspected postoperative complications after VHR. CT findings consistently allow diagnosis of abdominal wall abscesses and fistulas, haematomas with or without active bleeding, bowel obstruction, peritonitis, and recurrent hernias. Familiarity with early post-surgical CT studies is warranted to avoid misinterpretation of the expected postoperative appearance.
